# Reduced renal function may explain the higher prevalence of hyperuricemia in older people

**DOI:** 10.1038/s41598-020-80250-z

**Published:** 2021-01-14

**Authors:** Yutang Wang, Wanlin Zhang, Tingting Qian, Hui Sun, Qun Xu, Xujuan Hou, Wenqi Hu, Guang Zhang, Grant R. Drummond, Christopher G. Sobey, Fadi J. Charchar, Jonathan Golledge, Guang Yang

**Affiliations:** 1 Discipline of Life Sciences, School of Science, Psychology and Sport, Federation University Australia, Ballarat, VIC 3350 Australia; 2 Department of Gerontology, The First Affiliated Hospital of Shandong First Medical University, 16766 Jingshi Road, Jinan, 250014 Shandong Province China; 3 Department of Geriatric Medicine, School of Clinical Medicine, Weifang Medical University, Weifang, Shandong Province China; 4 The Health Physical Examination Center, The First Affiliated Hospital of Shandong First Medical University, Jinan, Shandong Province China; 5 Centre for Cardiovascular Biology and Disease Research and Department of Physiology, Anatomy and Microbiology, School of Life Sciences, La Trobe University, Melbourne, VIC Australia; 6 Queensland Research Centre for Peripheral Vascular Disease, College of Medicine and Dentistry, James Cook University, Townsville, QLD Australia; 7 Department of Vascular and Endovascular Surgery, The Townsville University Hospital, Townsville, QLD Australia

**Keywords:** Medical research, Risk factors

## Abstract

This study aimed to investigate the contribution of renal dysfunction to enhanced hyperuricemia prevalence in older people. A cohort of 13,288 Chinese people aged between 40 and 95 years were recruited from January to May 2019. Serum uric acid concentration and estimated glomerular filtration rate [eGFR] were measured. The associations between age or eGFR and serum uric acid or hyperuricemia were analyzed using linear or binary logistic regression adjusting for risk factors. Uric acid concentration and prevalence of hyperuricemia were greater in older participants. Adjustment for reduced renal function (eGFR < 60 mL/min/1.73 m^2^) eliminated the associations between older age and higher uric acid concentration and between older age and higher prevalence of hyperuricemia diagnosis, whereas adjustment for other risk factors did not change those associations. Lower eGFR was associated with higher uric acid concentration both before (β = − 0.296, *P* < 0.001) and after adjustment for age (β = − 0.313, *P* < 0.001). Reduced renal function was associated with hyperuricemia diagnosis both before (odds ratio, OR, 3.64; 95% CI 3.10–4.28; *P* < 0.001) and after adjustment for age (adjusted OR, 3.82; 95% CI 3.22–4.54; *P* < 0.001). Mean serum uric acid and prevalence of hyperuricemia were higher in people with eGFR < 60 mL/min/1.73 m^2^ than those with eGFR ≥ 60 mL/min/1.73 m^2^. The prevalence of reduced renal function increased with older age (*P* < 0.001). This study suggests that reduced renal function can explain the increased uric acid levels and hyperuricemia diagnoses in older people.

## Introduction

Uric acid is the end product of metabolic breakdown of purine compounds^1^. Its concentration in serum is a balance between its production, which is catalyzed by xanthine oxidase^2^, and excretion mainly via the urine^3^. High circulating uric acid (hyperuricemia) has been reported to be associated with many health problems such as hypertension^4,5^, metabolic syndrome^6^, coronary artery disease^7^, stroke^8^ and preeclampsia^9^ and kidney disease^10–12^ which may be associated with the pro-inflammatory effect of uric acid^13^.

A number of factors can affect serum uric acid levels. For example, renal dysfunction can impair the excretion of uric acid and thus can increase serum uric acid level leading to hyperuricemia^14^. Dietary and behavioral factors including higher meat consumption, drinking, smoking, less sleep, and sedentary lifestyle can raise serum uric acid levels^14–18^. In addition, male sex, higher BMI, higher total cholesterol, higher triglyceride, hypertension, and diabetes are common risk factors for hyperuricemia^14–17^.

It has been reported that serum uric acid and the prevalence of hyperuricemia increase in aged Chinese people^19,20^. Similar findings are also reported in people from other countries such as Austria^21^ and United States^22^. However, the reasons underlying these observations are unknown^23^. This study aimed to investigate the contribution of renal dysfunction to higher uric acid levels and enhanced hyperuricemia prevalence in older people using a large Chinese cohort (N = 13,288). We hypothesized that older age is associated with both higher uric acid levels and higher prevalence of hyperuricemia, and that adjustment for reduced renal function would eliminate those positive associations.

## Results

### The characteristics of the cohort

A total of 13,288 participants including 7782 men and 5506 women aged 40–95 years were included (Table 1). Among these participants, 13.9% were hyperuricemic and 5.5% had reduced renal function (eGFR < 60 mL/min/1.73 m^2^). No participants were on uric acid lowering or dialysis therapies.

**Table 1:** Characteristics of the study participants.

	Total	Men	Women	*P* value^a^
Sample size	13,288	7.782	5.506	NA
Age, median (IQR), y	53 (46–61)	53 (46–61)	53 (46–61)	0.089
Serum uric acid, Median (IQR), mg/dL	5.21 (4.36–6.18)	5.81 (5.04–6.67)	4.44 (3.80–5.11)	< 0.001
Hyperuricemia^b^, %	13.9	17.9	8.2	< 0.001
Hypertension^c^, %	40.4	46.2	32.2	< 0.001
eGFR, median (IQR), mL/min/1.73 m^2^	79.5 (71.1–89.0)	78.6 (70.5–87.3)	81.0 (72.0–90.9)	< 0.001
Reduced renal function^d^, %	5.5	5.9	4.9	0.018
BMI, median (IQR), kg/m^2^	25.1 (23.0–27.3)	25.8 (24.0–27.8)	23.9 (22.0–26.1)	< 0.001
FPG, median (IQR), mmol/L	5.25 (4.89–5.83)	5.35 (4.95–6.01)	5.13 (4.80–5.59)	< 0.001
TG, median (IQR), mmol/L	1.25 (0.89–1.79)	1.36 (0.97–1.94)	1.13 (0.81–1.58)	< 0.001
TC, median (IQR), mmol/L	4.85 (4.29–5.48)	4.77 (4.23–5.37)	4.99 (4.40–5.65)	< 0.001

BMI, body mass index; eGFR, estimated glomerular filtration rate; IQR, interquartile range; NA, not applicable; FPG, fasting plasma glucose; TC, total cholesterol; TG, triglyceride.

^a^Compared between men and women. The Mann Whitney U was used for continuous variables and Fisher’s exact test was used for categorical variables.

^b^Hyperuricemia was defined as uric acid ≥ 7 mg/dL for men and ≥ 6 mg/dL for women.

^c^Hypertension was defined as systolic blood pressure ≥ 140 mm Hg or diastolic blood pressure ≥ 90 mm Hg.

^d^Reduced renal function was defined as eGFR < 60 mL/min/1.73 m^2^.

### Uric acid concentration and prevalence of hyperuricemia are greater in older participants

**Figure 1:**
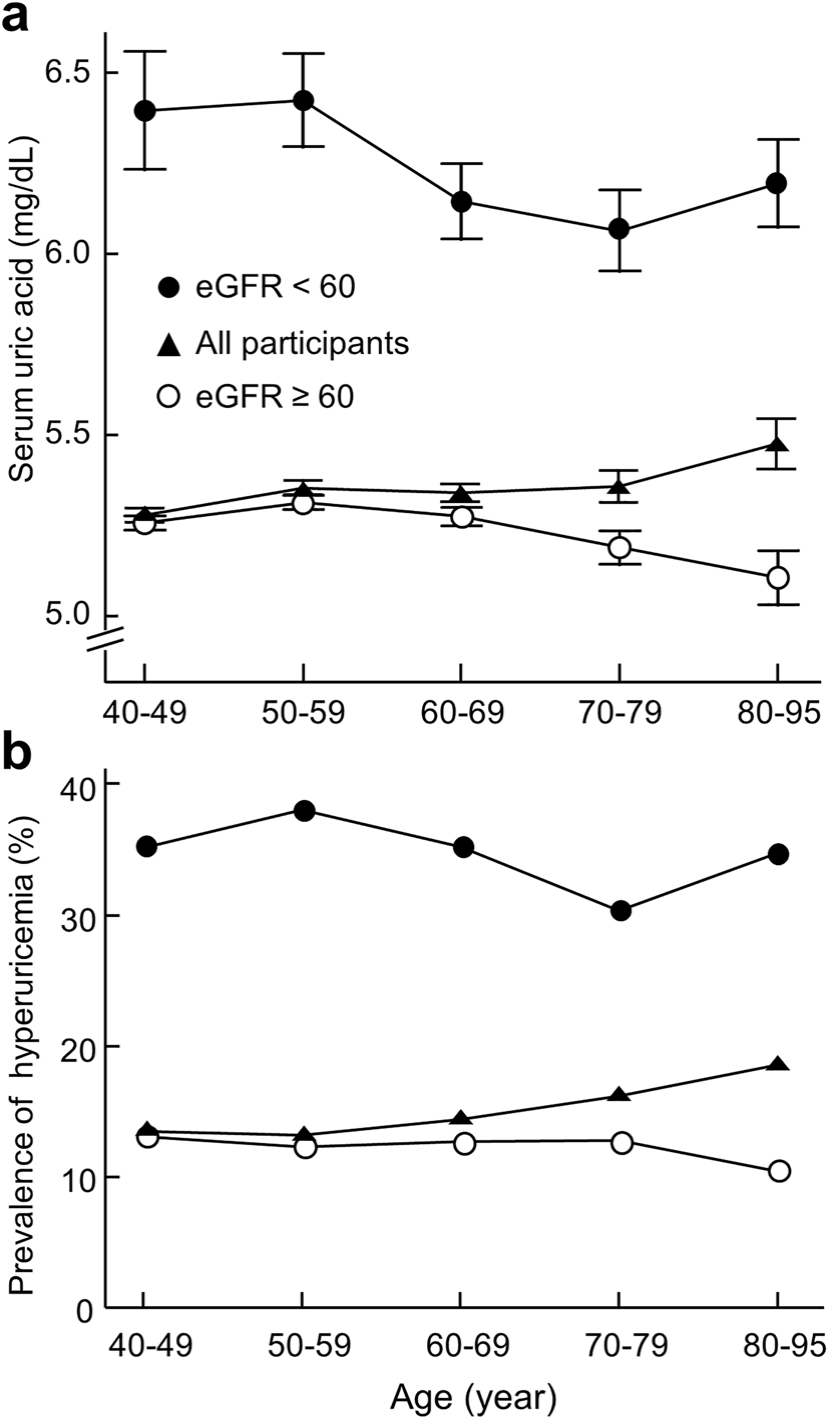
Uric acid concentration and prevalence of hyperuricemia across age groups in 13,288 participants. (**a**) Uric acid concentration and (**b**) prevalence of hyperuricemia across age groups in all participants (filled triangles) or participants with (filled circles) or without reduced renal function (open circles). Reduced renal function was defined as estimated glomerular filtration rate (eGFR) < 60 mL/min/1.73 m^2^. Data represent mean ± SE. The difference in the prevalence of hyperuricemia among age groups was analyzed by the chi-square test. *P* = 0.006, *P* = 0.703, and *P* = 0.631 for all participants, participants with or without reduced renal function, respectively.

Older age was associated with higher serum uric acid (β = 0.026, *P* = 0.003, Table 2) and higher prevalence of hyperuricemia diagnosis (OR, 1.007; 95% CI 1.002–1.011; *P* < 0.005, Table 3). A 10-year increase in age of participants was associated with a mean increase in uric acid of 0.03 mg/dL and with a 7% increased risk of hyperuricemia. Mean serum uric acid increased from 5.28 mg/dL in 40–49 years of age to 5.47 mg/dL in the 80–95 years age group (Fig. 1a) and the prevalence of hyperuricemia increased from 13.5% in the 40–49 years of age group to 18.6% in the 80–95 years age group (Fig. 1b).

**Table 2:** Association between age and serum uric acid using linear regression analysis.

	β	*P* value
Univariable	0.026	0.003
Multivariable^a^	− 0.018	0.048
Multivariable^b^	0.026	< 0.001
Multivariable^c^	− 0.010	0.187

^a^Adjusted for reduced renal function (estimated glomerular filtration rate rate < 60 mL/min/1.73 m^2^).

^b^Adjusted for sex, fasting glucose, hypertension, body mass index, total cholesterol, and triglycerides.

^c^Adjusted for sex, fasting glucose, hypertension, body mass index, total cholesterol, and triglycerides in addition to reduced renal function.

**Table 3:** Association between age and hyperuricemia using binary logistic regression analysis.

	OR	*P* value
Univariable	1.007 (1.002–1.011)	0.005
Multivariable^a^	0.996 (0.996–0.991)	0.114
Multivariable^b^	1.011 (1.005–1.016)	< 0.001
Multivariable^c^	1.001 (0.995–1.006)	0.810

^a^Adjusted for reduced renal function (estimated glomerular filtration rate rate < 60 mL/min/1.73 m^2^).

^b^Adjusted for sex, fasting glucose, hypertension, body mass index, total cholesterol, and triglycerides.

^c^Adjusted for sex, fasting glucose, hypertension, body mass index, total cholesterol, and triglycerides in addition to reduced renal function.

### The effect of renal function on associations between age with uric acid concentration and hyperuricemia

After adjusting for reduced renal function, older age was no longer associated with higher serum uric acid; rather it was associated with a lower serum uric acid concentration (β = − 0.018, *P* = 0.048, Table 2). Mean uric acid in people without reduced renal function decreased from 5.26 mg/dL in the 40–49 years age group to 5.11 mg/dL in the 80–95 years age group (Fig. 1a). Adjusting for other risk factors for hyperuricemia did not change the association between older age and higher serum uric acid (Table 2). In addition, after adjusting for reduced renal function, older age was no longer associated with a higher risk for hyperuricemia (*P* = 0.114, Table 3). After the exclusion of reduced renal function, the prevalence of hyperuricemia no longer increased with older age (Fig. 1b). Adjusting for other risk factors for hyperuricemia did not change the association between older age and enhanced prevalence of hyperuricemia diagnosis (Table 3). These results suggest that reduced renal function explains the increased serum uric acid concentration and prevalence of hyperuricemia in aged people.

### Reduced renal function is associated with higher uric acid concentration and higher hyperuricemia diagnosis independent of age

eGFR was negatively associated with uric acid concentration across all participants (β = − 0.296, *P* < 0.001). A 10 mL/min/1.73 m^2^ decrease in eGFR of participants was associated with a mean increase of 0.28 mg/dL in uric acid. The association was independent of age alone (β = − 0.313, *P* < 0.001) or age together with other risk factors for hyperuricemia including sex, fasting glucose, hypertension, body mass index, total cholesterol, and triglycerides (β = − 0.243, *P* < 0.001). Consistently, people with reduced renal function had higher serum uric acid compared to those with preserved renal function (median [interquaricle range], 6.15 [5.15–7.14] mg/dL versus 5.17 [4.32–6.12] mg/dL, *P* < 0.001).

When eGFR was treated as a continuous variable, higher eGFR was associated with a lower risk for hyperuricemia diagnosis (OR, 0.956; 95% CI 0.952–0.959; *P* < 0.001), such that a 10 mL/min/1.73 m^2^ increase in eGFR was associated with a 44% decreased risk of hyperuricemia. The association between higher eGFR and lower prevalence of hyperuricemia diagnosis remained after adjusting for age alone (OR, 0.987; 95% CI 0.982–0.992; *P* < 0.001) or age together with other risk factors for hyperuricemia including sex, fasting glucose, hypertension, body mass index, total cholesterol, and triglycerides (OR, 0.955; 95% CI 0.950–0.959; *P* < 0.001).

When eGFR was treated as a categorical variable (< 60 or ≥ 60 mL/min/1.73 m^2^), reduced renal function (< 60 mL/min/1.73 m^2^) was associated with higher prevalence of hyperuricemia diagnosis (OR, 3.64; 95% CI 3.10–4.28; *P* < 0.001). The association remained after adjustment for age alone (OR, 3.82; 95% CI 3.22–4.54; *P* < 0.001) or age together with other risk factors for hyperuricemia including sex, fasting glucose, hypertension, body mass index, total cholesterol, and triglycerides (OR, 3.37; 95% CI 2.82–4.04; *P* < 0.001), meaning that reduced renal function increased the prevalence of hyperuricemia diagnosis independent of age.

Further sub-analysis was conducted in which eGFR was divided into three categories, *i.e.* ≥ 60, < 60 and ≥ 30, and < 30 mL/min/1.73 m^2^. Compared to people with an eGFR ≥ 60 mL/min/1.73 m^2^, people with an eGFR between 30 to 60 mL/min/1.73 m^2^ had an increased risk of hyperuricemia (OR, 3.57; 95% CI 3.03–4.21; *P* < 0.001), and people with an eGFR < 30 mL/min/1.73 m^2^ had a much higher risk of hyperuricemia (OR, 7.74; 95% CI 2.98–20.09; *P* < 0.001). The association remained after adjustment for age, sex, fasting glucose, hypertension, body mass index, total cholesterol, and triglycerides.

**Figure 2:**
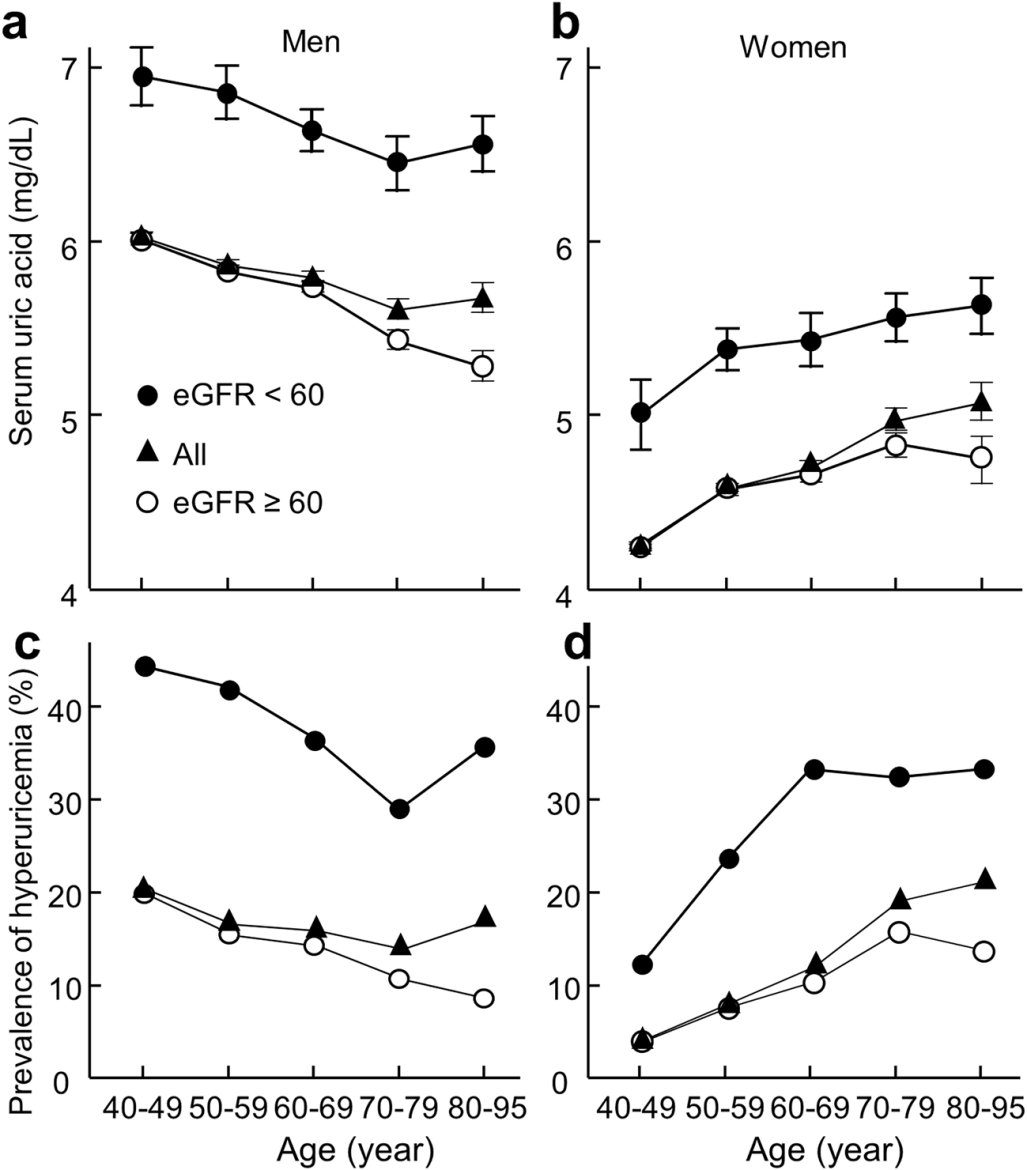
Uric acid concentration and prevalence of hyperuricemia across age groups in 7782 men or 5506 women. Uric acid concentration (**a,b**) and prevalence of hyperuricemia (**c,d**) in men (**a,c**) or women (**b,d**) across age groups. The participants in each gender were grouped as all participants (triangles) or participants with (filled circles) or without reduced renal function (open circles). Reduced renal function was defined as estimated glomerular filtration rate (eGFR) < 60 mL/min/1.73 m^2^. Data represent mean ± SE. The difference in the prevalence of hyperuricemia among age groups was analyzed by the chi-square test. *P* < 0.001, *P* = 0.163, and *P* < 0.001 for all men, men with or without reduced renal function, respectively; and *P* < 0.001, *P* = 0.222, and *P* < 0.001 for all women, women with or without reduced renal function, respectively.

Gender-based sub-analysis showed that eGFR (as a continuous variable) was negatively associated uric acid concentration in both men and women before (β = − 0.285, *P* < 0.001 for men; β = − 0.308, *P* < 0.001 for women) and after adjustment for age, fasting glucose, hypertension, body mass index, total cholesterol, and triglycerides (β = − 0.283, *P* < 0.001 for men; β = − 0.271, *P* < 0.001 for women). Reduced renal function (< 60 mL/min/1.73 m^2^) increased both serum uric acid and prevalence of hyperuricemia in both men and women (Fig. 2). Binary logistic regression analysis confirmed that reduced renal function was associated with higher prevalence of hyperuricemia diagnosis in both men and women before (Men: OR, 3.02; 95% CI 2.47–3.68; *P* < 0.001. Women: OR, 5.46; 95% CI 4.12–7.28; *P* < 0.001) and after adjustment for age, fasting glucose, hypertension, body mass index, total cholesterol, and triglycerides (Men: adjusted OR, 3.49; 95% CI 2.81–4.35; *P* < 0.001. Women: adjusted OR, 2.84; 95% CI 2.06–3.92; *P* < 0.001).

### The prevalence of reduced renal function increases with older age

The prevalence of reduced renal function increased with age in the whole cohort and within each gender (Table 4).

**Table 4:** Prevalence of reduced renal function across age groups.

	Prevalence of reduced renal function,^a^ Number (%)					
	Age groups, years					*P* value^b^
	40–49	50–59	60–69	70–79	80–95	
All (N = 13,288)	88 (1.7)	147 (3.4)	184 (7.4)	171 (19.1)	138 (33.8)	< 0.001
Men (N = 7782)	63 (2.1)	104 (4.1)	109 (7.5)	97 (18.2)	84 (31.5)	< 0.001
Women (N = 5506)	25 (1.1)	43 (2.5)	75 (7.1)	74 (20.3)	54 (38.3)	< 0.001

^a^Reduced renal function was defined as estimated glomerular filtration rate < 60 mL/min/1.73 m^2^.

^b^Chi-square test was used to compare the difference among age groups.

## Discussion

This study demonstrates that serum levels of uric acid and the prevalence of hyperuricemia are associated with older age and this positive association disappears after adjusting for reduced renal function, but not for other risk factors for hyperuricemia. These results suggest that reduced renal function explains the higher uric acid levels and enhanced prevalence of hyperuricemia diagnosis in older people.

Renal dysfunction can impair the excretion of uric acid and increase its serum level leading to hyperuricemia^14^. The current study found that lower eGFR was associated with higher serum uric acid and higher hyperuricemia diagnosis, independent of age and other risk factors. Consistent with the literature report ^24^, this study found that the percentage of people with reduced renal function increased with age. Therefore, increased prevalence of reduced renal function appears to be the underlying mechanism of the increased prevalence of hyperuricemia in aged people.

It was usually thought that the increase in circulating uric acid would lead to a corresponding worsening of renal function. However, very recent evidence showed that uric acid-lowering therapy did not slow the decline in renal function in patients with chronic kidney disease^25^ or with type 1 diabetes^26^, nor did it produce benefits on kidney failure^27^, indicating that hyperuricemia is not a cause of chronic kidney disease. Instead, these reports and our study suggest that the progression of chronic kidney disease is the cause of the increase in circulating uric acid.

This study showed that circulating uric acid levels in people with preserved renal function showed a gender-dependent pattern: a gradual decrease over time in men aged over 40 years versus an increase in women during the transition from 40–49 years to 50–59 years. The underlying mechanism is unknown. Changes in sex hormones over time may result in this gender-dependent pattern. Estradiol can inhibit xanthine oxidase^28^, the key enzyme in the uric acid production pathway; whereas testosterone can stimulate the enzyme^29^. Uric acid-inhibitory estradiol decreases in women at menopause and uric acid-stimulatory testosterone in men decreases gradually after 40 years^30^. The pattern of changes in those sex hormones seems to mirror the pattern of changes in uric acid observed in our study. However, whether this explanation is true needs to be investigated in the future.

A strength of this study is the large sample size that included subjects spanning several decades of older people which allowed the analysis of the effect of reduced renal function on the association between older age and hyperuricemia. This study has several limitations. First, we did not investigate dietary or behavioral risk factors such as higher meat consumption, drinking, higher alcohol intake, smoking, less sleeping, and sedentary lifestyle^14–18^ which may raise serum uric acid levels of the participants. Second, the results in this study concern the inhabitants of eastern and central Asia, and future studies will clarify whether our results apply to people of other ethnicities, e.g. Caucasians.

In conclusion, this study found that higher prevalence of reduced renal function appears likely to be responsible for the increase in serum uric acid in aged people.

## Methods

### Subjects

A total of 13,288 participants aged 40 years or more underwent a health examination between January and May 2019 at the Health Physical Examination Center of the First Affiliated Hospital of Shandong First Medical University, Jinan, Shandong Province, China. This retrospective study complied with the Declaration of Helsinki and was approved, and the requirement for obtaining patient informed consent was waived, by the Research Ethics Committee of First Affiliated Hospital of Shandong First Medical University.

### Measurements and definitions

Blood pressure was measured in all participants by trained professionals using electronic sphygmomanometry. Blood pressure was measured in the right arm in the seated position with elbow and forearm resting on the armrest after the participant rested for 10 minutes^31,32^. Blood pressure was measured 2 times at 2-min intervals in all participants and mean systolic and diastolic blood pressure were recorded. Hypertension was defined as systolic blood pressure ≥ 140 mm Hg or diastolic blood pressure ≥ 90 mm Hg^33^.

Venous blood samples were collected after an overnight fast (≥ 12 h). Serum uric acid concentration was measured by the uricaseperoxidase method^34^. Hyperuricemia was defined as serum uric acid concentration ≥ 7 mg/dL in men or ≥ 6 mg/dL in women^35^. The estimated glomerular filtration rate (eGFR) was measured using an Olympus AU2700 automatic biochemical analyzer. eGFR ≥ 60 mL/min/1.73 m^2^ was regarded as conserved kidney function and eGFR < 60 mL/min/1.73 m^2^ was regarded as reduced renal function^36^. Total cholesterol, triglycerides, and fasting plasma glucose were measured using an Olympus AU2700 automatic biochemical analyzer.

### Statistical analysis

All statistical analyses were performed using SPSS version 25.0 (IBM SPSS Statistics for Windows, Armonk, NY, International Business Machines Corporation). Age (Kolmogorov–Smirnov statistic, KS statistic, 0.094, *P* < 0.001), serum uric acid (KS statistic 0.035, *P* < 0.001), eGFR (KS statistic 0.038, *P* < 0.001), fasting plasma glucose (KS statistic 0.216, *P* < 0.001), total cholesterol (KS statistic 0.030, *P* < 0.001), body mass index (KS statistic 0.026, *P* < 0.001) and triglycerides (KS statistic 0.159, *P* < 0.001) were not normally distributed. Descriptive statistics were presented as median and interquartile range or numbers and percentages. The prevalence of hyperuricemia and reduced renal function were compared between participants of different ages by the chi-square test. Mann Whitney U and the Fisher’s exact test were used to test statistical differences between two groups. Only 6 participants were aged 90–95 years and they were combined with those aged 80–89 years to form an age group of 80–95 years. The associations between age and serum uric acid and between age and hyperuricemia were analyzed by linear regression and binary logistic regression, respectively, with or without adjusting for reduced renal function (eGFR < 60 mL/min/1.73 m^2^) or other risk factors for hyperuricemia including age, sex, fasting glucose, hypertension, body mass index, total cholesterol, and triglycerides. The associations between eGFR and serum uric acid and between reduced renal function and hyperuricemia were analyzed using linear regression and binary logistic regression, respectively, with or with adjusting for age or other risk factors for hyperuricemia. In a sub-analysis, 728 participants with reduced renal function (eGFR < 60 mL/min/1.73 m^2^) were excluded. Sub-analyses were also conducted where eGFR was divided into three categories (≥ 60, < 60 and ≥ 30, and < 30 mL/min/1.73 m^2^) or participants were grouped into men and women. A *P* value of < 0.05 was regarded as statistically significant.

## Data Availability

The datasets are available from the corresponding author on reasonable request.

## References

[1] Wu XW, Lee CC, Muzny DM, Caskey CT (1989). Urate oxidase: primary structure and evolutionary implications. Proc. Natl. Acad. Sci. USA.

[2] Mandal AK, Mount DB (2015). The molecular physiology of uric acid homeostasis. Annu. Rev. Physiol..

[3] Tsushima Y (2013). Uric acid secretion from adipose tissue and its increase in obesity. J. Biol. Chem..

[4] Qu LH, Jiang H, Chen JH (2017). Effect of uric acid-lowering therapy on blood pressure: systematic review and meta-analysis. Ann. Med..

[5] Johnson RJ (2013). What are the key arguments against uric acid as a true risk factor for hypertension?. Hypertension.

[6] Ford ES, Li C, Cook S, Choi HK (2007). Serum concentrations of uric acid and the metabolic syndrome among US children and adolescents. Circulation.

[7] Brand FN, McGee DL, Kannel WB, Stokes J, Castelli WP (1985). Hyperuricemia as a risk factor of coronary heart disease: the Framingham Study. Am. J. Epidemiol..

[8] Lehto S, Niskanen L, Rönnemaa T, Laakso M (1998). Serum uric acid is a strong predictor of stroke in patients with non-insulin-dependent diabetes mellitus. Stroke.

[9] Roberts JM (2005). Uric acid is as important as proteinuria in identifying fetal risk in women with gestational hypertension. Hypertension.

[10] Siu YP, Leung KT, Tong MK, Kwan TH (2006). Use of allopurinol in slowing the progression of renal disease through its ability to lower serum uric acid level. Am. J. Kidney Dis..

[11] Mazzali M (2001). Elevated uric acid increases blood pressure in the rat by a novel crystal-independent mechanism. Hypertension.

[12] Tsai CW, Lin SY, Kuo CC, Huang CC (2017). Serum uric acid and progression of kidney disease: a longitudinal analysis and mini-review. PLoS ONE.

[13] Braga TT (2017). Soluble uric acid activates the NLRP3 inflammasome. Sci. Rep..

[14] Qiu L (2013). Prevalence of hyperuricemia and its related risk factors in healthy adults from Northern and Northeastern Chinese provinces. BMC Public Health.

[15] Nakanishi N, Yoshida H, Nakamura K, Suzuki K, Tatara K (2001). Predictors for development of hyperuricemia: an 8-year longitudinal study in middle-aged Japanese men. Metabolism.

[16] Ni Q, Lu X, Chen C, Du H, Zhang R (2019). Risk factors for the development of hyperuricemia: a STROBE-compliant cross-sectional and longitudinal study. Medicine.

[17] Oliveira IO (2020). Uric acid is independent and inversely associated to glomerular filtration rate in young adult Brazilian individuals. Nutr. Metab. Cardiovasc. Dis..

[18] Raja S (2019). Frequency of hyperuricemia and its risk factors in the adult population. Cureus.

[19] Liu DM, Jiang LD, Gan L, Su Y, Li F (2019). Association between serum uric acid level and body mass index in sex- and age-specific groups in southwestern China. Endocr. Pract..

[20] Zhang Q, Lou S, Meng Z, Ren X (2011). Gender and age impacts on the correlations between hyperuricemia and metabolic syndrome in Chinese. Clin. Rheumatol..

[21] Zitt E, Fischer A, Lhotta K, Concin H, Nagel G (2020). Sex- and age-specific variations, temporal trends and metabolic determinants of serum uric acid concentrations in a large population-based Austrian cohort. Sci. Rep..

[22] Zhu Y, Pandya BJ, Choi HK (2011). Prevalence of gout and hyperuricemia in the US general population: the National Health and Nutrition Examination Survey 2007–2008. Arthritis Rheum..

[23] 23Multidisciplinary Expert Task Force on Hyperuricemia and Related Diseases. Chinese Multidisciplinary Expert Consensus on the Diagnosis and Treatment of Hyperuricemia and Related Diseases. *Chin. Med. J. (Engl)* **130**, 2473–2488 (2017). 10.4103/0366-6999.216416 PMC5684625 29052570

[24] Denic A, Glassock RJ, Rule AD (2016). Structural and functional changes with the aging kidney. Adv. Chron. Kidney Dis..

[25] Badve SV (2020). Effects of allopurinol on the progression of chronic kidney disease. N. Engl. J. Med..

[26] Doria A (2020). Serum urate lowering with allopurinol and kidney function in type 1 diabetes. N. Engl. J. Med..

[27] 27Chen, Q. *et al.* Effect of urate-lowering therapy on cardiovascular and kidney outcomes: a systematic review and meta-analysis. *Clin. J. Am. Soc. Nephrol.* (2020). 10.2215/CJN.05190420 PMC7646244 33055192

[28] Huh K, Shin US, Choi JW, Lee SI (1994). Effect of sex hormones on lipid peroxidation in rat liver. Arch. Pharmacal. Res..

[29] Olatunji LA, Areola ED, Badmus OO (2018). Endoglin inhibition by sodium acetate and flutamide ameliorates cardiac defective G6PD-dependent antioxidant defense in gestational testosterone-exposed rats. Biomed Pharmacother.

[30] Walther A, Philipp M, Lozza N, Ehlert U (2016). The rate of change in declining steroid hormones: a new parameter of healthy aging in men?. Oncotarget.

[31] Clark CE, Taylor RS, Shore AC, Ukoumunne OC, Campbell JL (2012). Association of a difference in systolic blood pressure between arms with vascular disease and mortality: a systematic review and meta-analysis. Lancet.

[32] Cheng W (2017). The association between serum uric acid and blood pressure in different age groups in a healthy Chinese cohort. Medicine.

[33] Chobanian AV (2003). Seventh report of the joint national committee on prevention, detection, evaluation, and treatment of high blood pressure. Hypertension.

[34] Domagk GF, Schlicke HH (1968). A colorimetric method using uricase and peroxidase for the determination of uric acid. Anal. Biochem..

[35] Guo L (2014). Interpretation of the Chinese expert consensus: Recommendations for diagnosis and treatment of asymptomatic hyperuricemia complicated with cardiovascular diseases. J. Transl. Int. Med..

[36] Stevens PE, Levin A (2013). Evaluation and management of chronic kidney disease: synopsis of the kidney disease: improving global outcomes 2012 clinical practice guideline. Ann. Intern. Med..

